# The Relationship Between Yoga and Spirituality: A Systematic Review of Empirical Research

**DOI:** 10.3389/fpsyg.2021.695939

**Published:** 2021-08-02

**Authors:** Barbara Csala, Constanze Maria Springinsfeld, Ferenc Köteles

**Affiliations:** ^1^Doctoral School of Psychology, ELTE Eötvös Loránd University, Budapest, Hungary; ^2^Institute of Health Promotion and Sport Sciences, ELTE Eötvös Loránd University, Budapest, Hungary; ^3^Middle European Interdisciplinary Master’s Programme in Cognitive Science, University of Vienna, Vienna, Austria

**Keywords:** yoga, spirituality, aspects of spirituality, well-being, health

## Abstract

**Objective:**

Both yoga practice and spirituality are associated with beneficial mental health outcomes. Within yoga research, however, spirituality is still a widely neglected area. The present systematic review aims to explore empirical studies, which do, in fact, investigate the relationship between yoga and spirituality in order to provide an overview and future directions for research on this topic. The review examines whether available empirical research supports an association between yoga practice and spirituality and, if so, which specific aspects of spirituality are associated with yoga practice.

**Methods:**

The systematic review followed the PRISMA guideline (Prospero registration number: CRD42020155043). Empirical studies written in English, German, or Hungarian language were selected from a database search in Google Scholar, PsycINFO, and Science Direct. A total of 30 studies met the final inclusion criteria.

**Results:**

According to the quantitative and qualitative studies reviewed, yoga practice seems to be positively associated with spirituality. This association concerns various aspects of spirituality, such as spiritual aspirations, a search for insight/wisdom, an integrative worldview, a sense of meaning and peace, faith, hope, compassion, and happiness within. To harness the potential spiritual benefits of yoga, regular practice appears to be essential. Yoga practitioners seem to have both physical and spiritual motives for practicing. At least in Western societies, however, physical intentions are more prevalent than spiritual ones. The meaning of spirituality for yoga practitioners is also discussed. Due to risk of bias of the majority of the reviewed studies, however, outcomes must be taken with caution.

**Conclusion:**

Yoga practice may be positively associated with several aspects of spirituality. For more evidence, further investigation of the topic is suggested. Particularly, we propose the inclusion of holistic forms of yoga practice and a comparison of Eastern and Western approaches to yoga.

## Introduction

In the past few decades, research on yoga has gained immense popularity. By now, there are numerous empirical studies confirming the beneficial effects of yoga practice with regard to a broad variety of indicators of physical and mental health (Raub, 2002; Büssing et al., 2012b; Park et al., 2014; Dwivedi and Tyagi, 2016; Govindaraj et al., 2016; Patwardhan, 2017). Yoga practice seems to be effective to improve various measures of physical fitness and to aid diverse physical health conditions, starting from injuries up to, e.g., cancer, severe cardiovascular or metabolic diseases (Raub, 2002; Ross and Thomas, 2010; Roland et al., 2011; Büssing et al., 2012b; Bhavanani, 2013; Dwivedi and Tyagi, 2016). Yoga can be a great tool for the prevention or treatment of lifestyle-related diseases (mainly caused by sedentary lifestyle, alcohol or tobacco consumption, overeating, and overweight) (Bhavanani, 2013; Dwivedi and Tyagi, 2016), and it is also a natural remedy for various problems, which occur in old age (Roland et al., 2011; Mooventhan and Nivethitha, 2017). In addition, numerous studies and reviews have reported on the effects of yoga practice on positive mental health, such as mindfulness, resilience, affect, happiness, well-being, satisfactions with life, self-compassion, and social relationships (Ross et al., 2013; Riley and Park, 2015; Hendriks et al., 2017; Domingues, 2018). Others have reported on its positive effects on various psychological or (neuro)psychiatric disorders, such as depression, fatigue, anxiety and anxiety disorders, eating disorders, or sleep problems (Balasubramaniam et al., 2012; Büssing et al., 2012b; Sengupta, 2012; Field, 2016; Mooventhan and Nivethitha, 2017).

Yoga as a philosophical framework and a set of different methods originated thousands of years ago in ancient India (Baktay, 1992; Iyengar, 1994). It comprises various physical, mental, moral, and spiritual practices aimed at improving holistic health, well-being, and self-awareness (Iyengar, 1994; Impett et al., 2006; Ross and Thomas, 2010). In Western societies and research, however, yoga is often being reduced to a form of physical exercise and stretching (MacDonald, 2013; Sarbacker, 2014; Park et al., 2018). Yoga classes and interventions differ considerably with respect to the inclusion of philosophical or spiritual elements (Rama et al., 1976; Cramer et al., 2016; Park et al., 2018). The majority of all forms of yoga practice in the West fall under the umbrella term of “hatha yoga,” which is a body-focused practice involving physical postures (asana), breathing exercises (pranayama), and other classical yoga components, such as relaxation and meditation techniques (Devereux, 1994; Park et al., 2018; Csala et al., 2020). The understanding of yoga as a holistic philosophical and methodological framework, particularly concerning its fundamental spiritual nature and objectives, is often missing. On the other hand, there is an extensive body of literature, indicating that spirituality is highly relevant to human functioning and health (see below) (MacDonald, 2013; Sarbacker, 2014).

Spirituality is a broad and vague construct, which can be approached from psychological, philosophical, transcendental-religious, and phenomenological perspectives. It is considered a universal, human-specific phenomenon (Piedmont and Leach, 2002; Emmons, 2006; Tomcsányi et al., 2011), characterized by a search for and a belief in something sacred beyond the material world (Hill and Pargament, 2003; Emmons, 2006). It also refers to the subjective experience of a sense of truth (Heelas and Woodhead, 2005), wholeness and openness to the infinite (Kelly, 1995). Even though the two concepts are not identical, originally, spirituality was not distinguished from religion (Emmons, 2006; Tomcsányi et al., 2011). While religion is typically an institutionalized and culturally accepted system of faith (Hill and Pargament, 2003; Emmons, 2006), spirituality refers to an autonomous, individual experience, which does not necessarily exclude religious endeavors (Koenig et al., 2001). In traditional yogic terminology, spirituality refers to the spirit or soul, i.e., the innermost core of every human being, which can also be interpreted as pure consciousness (Rama et al., 1976; Baktay, 1992; Satyananda Saraswati, 2013; Swartz, 2015). The ultimate goal of spiritual practices in yoga is the “realization of the oneness of all things” (Satyananda Saraswati, 2013, p. 18), namely the insight that individual consciousness is part of/the same as universal consciousness. This experience is often referred to as “self-realization,” “oneness,” “union,” or “highest state of consciousness” (Rama et al., 1976; Iyengar, 1994; Satyananda Saraswati, 2013; Swartz, 2015). From a scientific point of view, since spirituality is a multi-shaded and multilayered concept, there is no *one* widely accepted definition of it but several similar or complementary ones. Similarly, there is no one consented methodology for investigating spirituality, but various established approaches to assess specific aspects of it (Emmons, 2006; MacDonald, 2013; Oman, 2013). As mentioned earlier, empirical findings corroborate the favorable impact of many aspects of spirituality on physical and mental health (FETZER/NIA Working Group, 1999). Aspects such as spiritual experiences, connection/closeness to God, spiritual involvement, and religious/spiritual support are associated with lower levels of depressive symptoms, higher levels of subjective well-being, positive affect, self-esteem, personal growth, and life satisfaction (Bergin, 1997; Emmons, 2006; Powers et al., 2007; Wheeler and Hyland, 2008; Komninos, 2009). Studies with multidimensional measures of spirituality support these outcomes (FETZER/NIA Working Group, 1999; Geary, 2003; Klein et al., 2016). However, some aspects of spirituality are not obviously in correlation with better mental health. According to Pikó et al. (2011), for instance, spirituality as a connection to the transcendent or God (namely the “religious aspect” of spiritual well-being) has low or no correlation with depression and anxiety, while spirituality as a search for meaning of life, goals, and values (namely its “existential aspect”) makes one less prone to depression, anxiety, and substance abuse. Some spiritual beliefs may even lead to adverse coping (Hill and Pargament, 2003; Peterson and Seligman, 2004; Gall et al., 2005; Crowley and Jenkinson, 2009) or modern health worries (Köteles et al., 2016). In addition, possibly occurring spiritual struggles can lead to temporary distress, fear, anxiety, depressive symptoms, and decrease in quality of life (Kornfield, 1989; Hill and Pargament, 2003). These findings, however, do not necessarily contradict the above-mentioned beneficial health effects of spirituality (Cook et al., 2009).

To date, only a few studies have investigated the contribution of spirituality to the positive effects of yoga practice. In fact, scientific research on yoga rarely incorporates spiritual variables (Büssing et al., 2012a; MacDonald, 2013; Csala et al., 2020). MacDonald (2013) draws attention to the importance of including spirituality in the scientific research of yoga. On the one hand, he suggests investigating the effects of yoga practice on spirituality and, on the other hand, examining a potential mediator or moderator role of spirituality in the relationship between yoga practice and other positive mental health measures.

To foster this proposal, the aim of the present systematic review was to assess the available empirical research on the relationship between yoga and spirituality in order to provide an overview of existing findings and highlight future directions for investigating this topic. Of particular interest was, whether empirical findings indicate a positive association between yoga practice and spirituality, and, furthermore, to explore which aspects of spirituality are associated with yoga practice.

## Methods

### Protocol

We followed the preferred reporting items for the systematic reviews and meta-analyses (PRISMA) guideline (Liberati et al., 2009). The protocol was registered in the PROSPERO international prospective registry of systematic reviews (registration number: CRD42020155043; date of registration: November 1, 2019).

### Literature Search

A systematic literature search was completed in November 2019. The searched databases were Google Scholar, PsycINFO, and Science Direct (incl. PsycARTICLES). Included were all articles which contained the terms “yoga, spirituality,” “yoga, spiritual,” or “yoga, spirit” in the title or the abstract. The search was not restricted in terms of the publication period. Additional articles from other sources were identified by examining the references of the studies obtained through the database search. Moreover, studies known by the authors from other sources and one previous article of the authors (BC and FK) were included.

### Inclusion and Exclusion Criteria

Included were empirical studies (cross-sectional and longitudinal/intervention studies) with sound methodology (that is, with clear methodological descriptions and adequate statistical or qualitative analyses; for quantitative studies, in best cases randomized, controlled studies) published in peer-reviewed scientific journals in either English, German, or Hungarian language. Reviews, meta-analyses, conference papers, books, book chapters, theses, and dissertations were excluded. No restriction was made to gender, nationality, or health status of the studied sample. Concerning age, we considered studies with individuals over 16 years; however, all of the full-text original articles included adult samples.

### Study Selection

**FIGURE 1:**
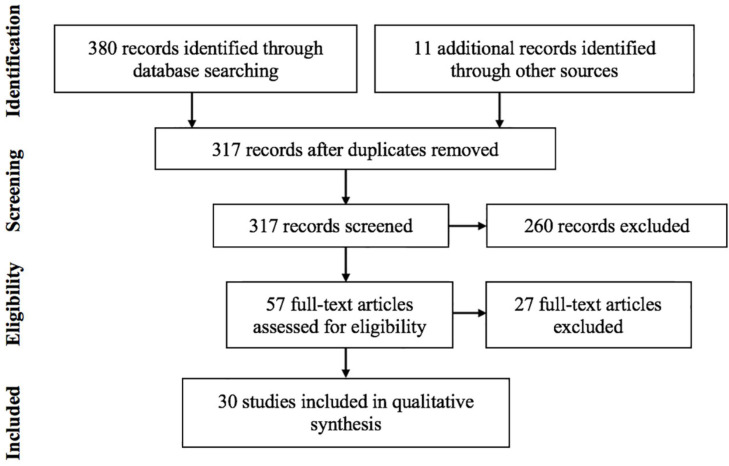
Selection of studies based on the PRISMA method.

The literature search yielded 380 records from the three databases and 11 records through other resources. After removing duplicates, the remaining 317 titles and abstracts were prescreened by two independent reviewers (BC and CMS). About 260 records were removed because they were not original papers and/or they were written in other languages. The remaining 57 full-text articles were assessed for eligibility by the same two independent reviewers (BC and CMS). From these, 27 full-text articles were excluded for one or more of the following reasons; they were not empirical studies due to methodological issues or because the intervention and/or the data analysis were delineated extremely poorly or not at all with regard to spirituality. Disagreements between evaluations were resolved by consensus; further problematic issues were resolved by consulting the third author (FK). According to the final screening, 30 articles met the inclusion criteria of the review (for details, see Figure 1).

### Data Extraction

Data of quantitative and qualitative studies were annotated into separate databases. Prespecified data were collected for each study. These included general classifiable information about the objectives, design, sample, spirituality measures, type of yoga practice/intervention, frequency of practice/intervention, the previous experience of participants with yoga, major findings, and the origin of the study. The databases were verified by the third reviewer (FK).

### Risk of Bias Assessment

Risk of bias was evaluated for randomized controlled studies and cross-sectional studies with quantitative approach. RCTs were assessed with the revised Cochrane risk-of-bias tool for randomized trials (RoB 2.0) (Higgins et al., 2011; Sterne et al., 2019). Five different domains are investigated with this measure: (1) a randomization process, (2) deviations from the intended intervention, (3) missing outcome data, (4) measurement of the outcome data, and (5) selective reporting of the results. Within each domain, a different number of questions are answered with either “yes,” “probably yes,” “probably no,” “no,” or “no information.” The outcome of the algorithm results in “low risk,” “some concern,” or “high risk” of bias for each domain and overall. For cross-sectional studies, the Joanna Briggs Institute (JBI) critical appraisal checklist for analytical cross-sectional studies was used (Ma et al., 2020; Moola et al., 2020). It consists of eight questions concerning sample, setting, measurement, confounding factors, and statistics. For each item, the answer indicates to what degree it is presented: either “yes,” “no,” “unclear,” or “not applicable.” None of these two measures were used as selection criteria for this review, but to assess possible risk of bias. Thus, percentages of the specific answers to the (JBI) critical appraisal checklist were calculated.

## Results

### General Data

All in all, 30 studies were included in the analysis. As shown in Supplementary Table 1 and Table 1, 25 of them applied quantitative methods and seven qualitative ones (that is, two studies applied both). Fifteen studies had cross-sectional and 15 longitudinal designs encompassing a yoga intervention. Out of the 25 quantitative studies, nine included a control group. Among those, three papers had a cross-sectional design, and six of them were intervention studies, applying randomization (RCT studies). One of the qualitative studies had a randomized design as well, involving two different control groups. All study samples consisted of participants above the age of 16 years, except for one; all consisted of only adults. None of the samples can be considered representative of the respective population. The majority of them investigated participants without a common reported health problem, whereas seven studies focused on diseased populations; five of them on cancer patients, one on veterans with severe mental illness, and one on patients with symptomatic paroxysmal atrial fibrillation. There were two other unique samples: one a group of prisoners and the other a group of Zen hospice volunteers.

**TABLE 1:** Summary of the articles with qualitative approach.

Article	Design	Sample size (yoga group)	Control group(s)	Sample condition, age, gender	Measurement tool	Type of yoga practice	Frequency of intervention/previous yoga experience	Major findings	Origin of the study
Dittmann and Freedman, 2009; Study 2.	Cross-sectional	*N* = 18	No	Regular yoga practitioners from yoga and fitness centers; mean age: NR, age between 23 and 62 years; 100% female	15-min recorded phone interview: how yoga practice affected spirituality, and how spirituality affected eating and body image attitudes	NR (postural yoga)	Average number of years of yoga practice: 12.1; at least 1 h/week	Positive changes in sense of spirituality; yoga is a method for spiritual growth	United States
Griera, 2017	Longitudinal	*N* = 56	No	Prisoners (time spent in prison *M* = 2.25 years); mean age: 38.2 years; 16% female	Surveys (*N* = 56) and interviews (*N* = 25) of the impact, meaning, and implications of yoga practice in prison	Holistic program: Kundalini yoga based physical practice, and suggestion for yoga philosophy literature P, B, R, M, RB, Ma	”yoga quarantine”: 40 days, 2–3 h/day; “yoga intensive”: 2 months, 2 h/three times a week	Yoga is different from other form of movements: (1) Divine and transcendent experiences (feeling of freedom, silence, connectedness) (2) Brings an expansive spiritual semantic framework of life, it is a means of self-improvement (3) Offers a transcendent layer of reality	Europe (Spain)
Hasselle-Newcombe, 2005	Cross-sectional	*N* = 188	No, but compared to data of general United Kingdom population	Regular Iyengar yoga practitioners; mean age: 47 years; 84% female	17 pages long questionnaire: multiple choice, Likert scale questions and qualitative comments on aspects of yoga practice which are not directly relevant to spirituality, reasons why people begin and continue to practice yoga, perceived effects of yoga	Iyengar yoga (postural yoga)	Number of years of yoga practice: between 11 and 15; Average amount of yoga practice: 1 class/week + home practice 3–5 h/week	Long-term practitioners are no more likely to have a spiritual interest in their practice than beginners; yoga gives sense of meaning to life in 85% of practitioners; 83% of yoga practitioners have a spiritual life compared to 45% of the general United Kingdom population; concept of God associated with spirituality and not religiosity; spirituality: experience of ‘imminence,’ inward looking, mundane, but very personal experiences of holiness	Europe (United Kingdom)
Lewis, 2008	Cross-sectional	*N* = 9	No	Regular vinyasa yoga practitioners; mean age: NR; gender: NR	Loosely structured interviews with a focus on motivational factors for yoga practice	Vinyasa yoga (postural yoga)	NR	Enmeshed social, psychological, physical and spiritual meanings and motivations to continue yoga practice; motivation changes with time: originally to achieve the perfect ‘yoga body,’ later deeper mental and spiritual motivation	United States
Ness and Briles, 2016	Cross-sectional	*N* = 11	No	Yoga instructors; mean age: NR; 82% female	In-depth, semi-structured interviews about the traditional spirituality of yoga	Trained yoga teachers in various hatha yoga styles	NR	Lack of spirituality in westernized yoga; importance of meditation and yoga philosophy for spiritual progress; practice is the key to entering into yoga spirituality	United States
Pandya, 2018; Part 2.	Longitudinal	*N* = 19	No	Auroville spiritual community residents; Post intervention mean age: 44 years; 31,6% female	In-depth interview: open-ended questions about life and existence, aspirations, fears and anxieties, and spiritual experiences	Integral yoga of Sri Aurobindo LS	10 years, NR	Increased level of existential thinking, spiritual inclinations and integrative worldview after 10 years of residence; important aspects: philosophical teachings, consecration, meditation, prayer, and social support	India
Wahlström et al., 2018*	Longitudinal	*N* = 12	*N* = 44, relaxation group *N* = 44, only standard treatment	Patients with symptomatic paroxysmal atrial fibrillation; mean age: 63.5 years; 41.6% female	Individual semi-structured interviews on how participants experienced therapeutic yoga	MediYoga (therapeutic yoga derived from Kundalini yoga) P, R, M	12 weeks, weekly 1 h + encouraged to perform MediYoga at home with CD-record	Yoga participants’ perceptions and experiences: (1) A sense of existence and presence – experience of more inner peace, (2) Increased awareness of connection of physical and mental functions	Europe (Sweden)

NR, not reported; P, posture; B, breathing exercise; R, relaxation; M, meditation; LS, lifestyle advice; RB, reading books, manuals, or other teachings; Ma, mantras. *Study with randomized design.

Concerning risk of bias, assessment of RCTs (*N* = 6) indicated overall some concerns for three studies and high risk of bias for three others. High or some concerns occurred due to the randomization process or missing outcome data (large% of dropouts). Measurement of the outcome and the selection of reported results showed some concerns for all studies. Deviations from intended intervention showed low risk for each case (see Appendix 1 for details). Risk of bias assessment of cross-sectional studies with quantitative approach (*N* = 12) resulted in four articles with 50% or less than 50% of “yes” answers, indicating high risk with an insufficient sample and setting reporting, and lack of identification of confounding factors. No answers with “not clear” or “not applicable” occurred for any of the studies. Five further papers were characterized by 70 or 62.5% of “yes” answers and, therefore, by some concerns. The main insufficiency in these studies was the omission of confounding factors. Three studies, in turn, stated and investigated confounding factors, resulting in 75 or 100% of “yes” answers, which indicate low risk of bias. Furthermore, all studies used objective, standard criteria for measurement of the condition, measured outcomes in a valid and reliable way, and used appropriate statistical analyses (see Appendix 2). Summing up, RCT studies show some to high risk of bias, whereas cross-sectional studies in total raise some concerns. Because the measurement tools we applied detected risks of bias, the outcomes of the respective RCTs and cross-sectional studies must be taken with caution.

In line with some of the above-mentioned shortcomings, not all studies explicitly stated the previous experience of the participants with yoga. However, 18 characterized them as regular yoga practitioners and three as beginners. Some of the studies do not provide very detailed information on the type of yoga practice; five of them do not report the exact type at all. We can, nonetheless, assume that they investigated some type of postural yoga (hatha yoga). Including these five articles, 21 studies investigated different types of hatha yoga, making it the prevailing type of researched yoga practice. Among them, two papers specifically examined Iyengar yoga, and one paper reported on the views of vinyasa yoga practitioners. Moreover, four studies applied hatha yoga-based restorative classes (postural yoga designed for diseased populations). In total, only five studies included an intensive holistic (integral) yoga practice. It can be concluded that most of the studies, more precisely 25 out of 30, investigated a form of hatha yoga. This indicates that, in empirical research, yoga is mainly studied as a form of mind-body exercise (Mehling et al., 2011; Park et al., 2018).

In the next paragraph, we discuss the relevance of the origin of the reviewed studies. Thereafter, outcomes are presented by thematic topics (aspects of spirituality, regularity of practice, intention of practice, meaning of spirituality for yoga practitioners, and other important aspects of yoga and spirituality), delineating firstly quantitative and then qualitative findings for each topic. This is often followed by a general discussion of the investigated topic. In the last paragraph of this section, we describe the spirituality questionnaires applied by the reviewed papers.

### Origin of the Study

Research on yoga and spirituality among Western scholars very likely differs from research in more traditional settings. This can be attributed to the fact that yoga practice is less spiritually rooted in Western societies than, for instance, in India (Singleton, 2010; Ness and Briles, 2016). Among the reviewed studies, six were conducted in India and 22 in Western countries (Europe or United States). The two remaining studies were conducted in Iran and Japan. Out of the six Indian studies, three were conducted by the same author and involved very intense holistic yoga programs; however, not each of them was described in detail (Pandya, 2017, 2018, 2019). Among the remaining three, scope and theoretical background of two other Indian studies were only vaguely established, and the type of the investigated yoga practice was not reported (Ahmad and Imtiaz, 2016; Nandeesh et al., 2016). In comparison, the Western studies provided more detailed accounts of their methodologies. However, only two (Büssing et al., 2012a; Griera, 2017) out of the 20 Western studies investigated holistic yoga programs, including philosophical and spiritual teachings. One Western study (Smith et al., 2011) compared two types of yoga groups: one group included yogic ethical guidelines, while the other was yoga as exercise. Also, in two other studies (Danhauer et al., 2008, 2009), yoga practice included one yogic ethical principal (namely ahimsa, that is, non-violence). This confirms the previously made statement that yoga practice in the West often misses spiritual teachings and is characterized as a mere form of physical activity or a mind-body exercise (Rama et al., 1976; Cramer et al., 2016; Park et al., 2018). According to in-depth, semi-structured interviews with yoga instructors, westernized yoga lacks spirituality, that is, most classes are devoid of spiritual teachings. This is mostly due to cultural barriers, lack of knowledge, loss of holistic perspective, and a requirement of being religiously or culturally neutral and devoid of spiritual contents (Ness and Briles, 2016). Even so, it is important to note that spiritual experiences or interest can be evoked by purely physical yoga practice (Gaiswinkler and Unterrainer, 2016; Ness and Briles, 2016).

### Aspects of Spirituality

As mentioned above, even yoga practice without explicit spiritual teachings can affect spirituality (Ness and Briles, 2016). The vast majority of the reviewed studies reported positive results concerning the connection between yoga and spirituality. Out of 28, there was only one study that did not report such an association (Danhauer et al., 2008), and there were two studies that reported only very minor positive outcomes (Moadel et al., 2007; Lötzke et al., 2016). Most of the studies found favorable outcomes; some reported partially positive, partially null findings. Longitudinal studies reported an increase of different aspects of spirituality, such as conscious interactions and compassion, prayer or trust in God, and a search for insight/wisdom after 6 months of yoga practice (Büssing et al., 2012a). Improved levels of spiritual well-being characterized by a sense of meaning and peace (Danhauer et al., 2009), faith (Nakau et al., 2013), and hope (Smith et al., 2011) were found after several weeks of yoga interventions. Pandya (2017) also reported an enhanced level of spiritual well-being, as well as increased levels of spiritual experiences (such as the feeling of deep inner peace and harmony, and proximity to the divine), aspirations, existential thinking, a development of an integrative worldview (i.e., the view that the reality is complex, namely both scientific and spiritual at the same time) (Pandya, 2018, 2019), and a decreased level of existential anxieties (Pandya, 2018) after intense and holistic yoga programs. Another longitudinal study (Safara and Ghasemi, 2017) found that yoga positively affected all aspects of spiritual intelligence (a construct that involves spiritual and intelligence structures (Emmons, 2000), namely critical existential thinking, production of personal meaning, transcendental consciousness, and expanded state of consciousness. This result is supported by a cross-sectional study (Seena et al., 2017), showing higher levels of spiritual intelligence in all dimensions among yoga practitioners compared with controls. Yoga practitioners were also found to have lower fear of death (Scherwitz et al., 2006), enhanced spiritual health, a more positive outlook on life, as well as more faith and happiness within (Monk-Turner and Turner, 2010). In accordance with those, qualitative studies reported positive changes in the sense of spirituality among yoga practitioners, indicating that yoga is a method to achieve spiritual growth and an integrative worldview (Dittmann and Freedman, 2009; Pandya, 2018). Regular female yoga practitioners reported a more holistic and positive relation to their body due to yoga practice. They became more self-explorative and aware of many aspects of life, more tolerant, compassionate, and less judgmental (Dittmann and Freedman, 2009). One participant explained her experiences as “being in contact with the breath is my time to be with the divine and experience myself in my body as I am” (Dittmann and Freedman, 2009, p. 285). The improved sense of spirituality among the residents of an integral yoga community consisted in feeling presence of God, experiencing a connection with the divine and nature, and being spiritually touched by the beauty of creation. Sustainable development and a harmonious relationship with nature are highly important to them, since it is a way to reach the divine and self-transcendence (Pandya, 2018).

In contrast, three quantitative papers did not report considerable changes in spirituality (Moadel et al., 2007; Danhauer et al., 2008; Lötzke et al., 2016). It is important to note that all of them investigated women with cancer and involved 10–12-week-long yoga interventions. One of them found no changes in spiritual well-being measured by a sense of meaning/peace and faith (Danhauer et al., 2008). Although the second study (Lötzke et al., 2016) showed an increase in reflection, that is positive interpretation of disease, such an increase was also shown in the physical exercise control group. Moreover, it decreased back to an initial level after 3 months. The third of these studies (Moadel et al., 2007) found that a subsample of patients not receiving chemotherapy showed deterioration of spiritual well-being in the control compared with the yoga group. These results are not in accordance with two of the above-mentioned studies (Danhauer et al., 2009; Nakau et al., 2013), which also investigated cancer patients. Even though they applied similar intervention types and settings, they reported positive changes in spiritual well-being. The contradictory results might be explained by the severe health problem of the participants. Percentage of patients actively undergoing medical treatment during the intervention was higher in the three studies with null or minor outcomes (61, 100, and 48%) than in those with favorable results (34 and 0%). Thus, it is required that future research includes comparison studies of diseased populations at various stages of treatment and disease, as well as comparisons of healthy and diseased populations. Moreover, the contradictory results could indicate that the positive effects of such yoga interventions on spirituality arise after around 10–12 weeks of weekly practice. Thus, further studies with varying length and intensity of practice are needed to shed more light on this association.

Comparing and summing up the results of studies, which included yoga as a physical exercise versus the ones which involved it as a holistic practice, no obvious differences can be revealed, partly due to the heterogeneity of the investigations. However, it can be said that most of the studies, including hatha yoga as a physical exercise, showed promising outcomes concerning various aspects of spirituality. Importantly, Smith et al. (2011) found no difference between the integrated yoga group and the yoga as the exercise group; both of the yoga groups showed an increased level of hope compared with the no intervention control. Overviewing randomized control interventions, three papers reported almost null or partially positive findings (Moadel et al., 2007; Danhauer et al., 2009; Lötzke et al., 2016), while four studies reported clear beneficial outcomes concerning spiritual experiences, spiritual intelligence, a level of hope, a sense of existence, presence, and awareness of body-mind connectedness (Smith et al., 2011; Safara and Ghasemi, 2017; Wahlström et al., 2018; Pandya, 2019).

### Regularity of Practice

An interesting factor in the relationship between yoga and spirituality is the regularity of practice. On the one hand, perceived benefits of yoga and aspects of spirituality, such as meaning of life, gratitude, or transcendence, are associated with yoga experience (namely the number of years and the total lifetime hours practicing yoga) (Moliver et al., 2013; Ivtzan and Papantoniou, 2014; Baker et al., 2015). On the other hand, spirituality is most strongly associated with the frequency of current yoga practice (Moliver et al., 2013; Gaiswinkler and Unterrainer, 2016; Csala et al., 2017). Levels of spirituality, as well as perceived benefits of yoga, are higher among practitioners with more current practice (including class duration and frequency) (Baker et al., 2015; Gaiswinkler and Unterrainer, 2016). Pandya (2019) also states that the strongest predictor of positive outcomes in spirituality is regular (self-)practice. The results of a study with follow-up (Lötzke et al., 2016) also support the importance of regularity of practice. The level of reflection of yoga participants (i.e., positive spiritual interpretation of disease) increased at the end of a 12-week long yoga intervention but decreased back to the initial level after 3 months (Lötzke et al., 2016). In addition, results of a qualitative analysis (Ness and Briles, 2016) show that regular practice is a key component for yoga spirituality, and that going deeper into yoga practice (i.e., committed practice resulting in experiences of healing, connectedness, synchronicity, and more control) brings more benefits (Ness and Briles, 2016). Summarizing these outcomes, we can conclude that the regularity of yoga practice plays a highly important role in spirituality. According to a recent study (Szabolcs et al., 2021), the regularity of yoga practice is also positively associated with mindfulness, body awareness, and self-compassion. These empirical findings are in accordance with the experience and teaching of many Eastern movement practices that generally emphasize the importance of regular (often daily) practice (Ueshiba, 1984; Tihanyi et al., 2016).

### Intention of Practice

Six papers mentioned the intention of yoga practice in relation to spirituality. Two of them (Ivtzan and Jegatheeswaran, 2015; Park et al., 2016) explicitly focused on this topic. Regular hatha yoga practitioners in the United Kingdom reported greater initial and continued physical intentions (yoga as a form of physical exercise) than spiritual ones (spiritual development). However, their spiritual intentions increased over time, suggesting that hatha yoga practiced in the West can cultivate spirituality. Additionally, practitioners with spiritual intentions reported higher levels of both eudaimonic and hedonic psychological well-being (Ivtzan and Jegatheeswaran, 2015). Similarly, regular hatha yoga practitioners (both students and yoga teachers) in the United States reported that their primary reasons for adopting yoga practice were exercise, flexibility, and stress relief. However, for 23.5% of the yoga students and for 50.4% of the yoga teachers, spirituality became the primary reason for maintaining practice. Thus, spirituality was the top changed reason and also the most frequent reason among the newly acquired primary ones. Moreover, spirituality was an additional reason for maintaining practice for further 48% of the students and 49.6% of the teachers (Park et al., 2016). Another study (Quilty et al., 2013) conducted in the United States (more precisely, in Texas), with respondents who were enrolled in a 4-week beginner yoga program found that reasons to start or return to yoga practice were, primarily, general wellness (81%), physical exercise (80%), and stress management (73%), followed by seeking spiritual experiences (37%). About 73% of them endorsed yoga as a spiritual activity. A further cross-sectional study (Dittmann and Freedman, 2009) found that the level of spirituality is higher in women practicing with psycho-spiritual reasons compared with those who practiced primarily for physical or appearance reasons. Psycho-spiritual reasons encompassed self-knowledge, awareness and management of feelings, increase of mindfulness, and interest in spiritual and philosophical foundations of yoga. Physical or appearance reasons were physical exercise, strength, stretching/flexibility, and improving physical appearance. According to a qualitative study among a large sample of regular Iyengar yoga practitioners in the United Kingdom (Hasselle-Newcombe, 2005), 60% of the participants began their practice as an alternative form of exercise. Moreover, it was an important reason for continued practice in 85% of the total sample. Interestingly, flexibility gained the highest percentage (94%) as a reason to continue yoga practice. In contrast, only 48% of the participants began their yoga practice with an idea of spiritual development, and 47% reported that spiritual aspects played an important role for continuing practice. Consequently, long-term practitioners of this sample are no more likely to have a spiritual interest than beginners. Therefore, it is possible that practitioners with a preexisting orientation toward spirituality feel that their yoga practice is spiritual (Hasselle-Newcombe, 2005). However, this contradicts the abovementioned findings of Ivtzan and Jegatheeswaran (2015), as well as the outcomes of the loosely structured interviews with vinyasa yoga practitioners, which specifically focused on the motivation for practicing yoga (Lewis, 2008). According to these interviews, motivation of practice changes over time. The initial aim is often to achieve the “perfect yoga body,” but, subsequently, more profound mental and spiritual intentions appear. There are enmeshed social, psychological, physical, and spiritual meanings and motivations to continue yoga practice (Lewis, 2008). We can conclude that some of the beginner yoga practitioners have a preexisting spiritual orientation, and that continued hatha yoga practice appears to enhance spiritual interest and motivations. The lack of this latter result in the study of Hasselle-Newcombe (2005) may be explained by the fact that, among modern hatha yoga styles, Iyengar yoga is the most asana-centered with detailed work on individual poses. Iyengar yoga teachers generally avoid metaphysical comments and remain enigmatic on any explicit questions about spirituality. Nevertheless, the study found that physical practice facilitates spiritual experiences by providing an inward-looking, conscious personal experience of own body, breath, and mind of the practitioner, resulting in glimpses of the infinite or holiness and providing a sense of meaning of life (Hasselle-Newcombe, 2005).

All in all, it seems that physical intentions to practice yoga are more frequent than spiritual ones. Even so, spiritual motivations can increase over time and are associated with greater levels of spiritual and psychological well-being. These outcomes are, however, limited to Western societies since all of the studies, which investigated the topic, were conducted in Western countries. The intentions to practice might be different in India or other Eastern societies.

### Meaning of Spirituality for Yoga Practitioners

The results of three qualitative studies (Hasselle-Newcombe, 2005; Griera, 2017; Wahlström et al., 2018) give a deeper insight into what yoga and spirituality mean to practitioners. As reported by prisoners taking part in an intense yoga program, yoga is different from other forms of movement and sports (Griera, 2017). Yoga practice can evoke divine and transcendent experiences; it offers them a possibility to transcend the here and now, which elicits the feeling of freedom. Exhausting the body with intense exercise in conjunction with stretching, breathing techniques, and focused awareness can lead to silence of the mind. The participants described experiences of “collective energy,” “connectedness,” “self-awareness,” “flow,” and “inner state of consciousness.” Furthermore, yoga provided them with an expansive spiritual semantic framework of life. It became a means of self-improvement and self-transformation, and offered a transcendent layer of reality (Griera, 2017). Regular Iyengar yoga practitioners consider spirituality an alternative value to materialism. Spirituality, namely what they sense/perceive primarily through physical practice, is an experience of “imminence,” i.e., an inward looking, a mundane but very personal experience of holiness. About 85% of the practitioners stated that yoga gives a sense of meaning to their life, and 83% of the responders reported to have a spiritual life compared with 45% of the general United Kingdom population (Hasselle-Newcombe, 2005). According to the participants of a 12-week-long intervention, yoga practice gives a sense of existence and presence, which leads to more inner peace. Moreover, it increases the awareness of the connection between physical and mental processes (Wahlström et al., 2018).

It is also important to note that yoga practitioners describe themselves as either spiritual or spiritual and religious, but very unlikely as religious and not spiritual (Hasselle-Newcombe, 2005; Emmons, 2006). In contrast to institutionalized religion, yoga emphasizes the direct conscious experience of the practitioner. In consequence, it enables a personal understanding of the divine and God (Hasselle-Newcombe, 2005). The ultimate goal of yoga is to unite (or reunite) with the divine, i.e., the unchanging essence of life through individual practice (Iyengar, 1994; Hasselle-Newcombe, 2005). In line with this view, Ahmad and Imtiaz (2016) found that spirituality is not associated with avoidant and anxious attachment to God among yoga practitioners. Other reviewed studies, which applied questionnaires containing the term “religious” (e.g., religious orientation: prayer/trust in God, religious commitment, or religious/spiritual well-being) (Büssing et al., 2012a; Gaiswinkler and Unterrainer, 2016; Pandya, 2019) presumably had a permissive interpretation of it, namely one akin to spirituality. All of them reported favorable outcomes.

In summary, it can be said that among yoga practitioners, spirituality predominantly means an individual search for and an experience of the divine, which can give a feeling of inner silence, freedom, and connectedness. Spirituality also gives a meaning or framework of life, leading to increased awareness, self-improvement, and self-transformation.

### Other Important Aspects of Yoga and Spirituality

As mentioned in the introduction, both yoga and various aspects of spirituality are associated with beneficial mental health effects (FETZER/NIA Working Group, 1999; Emmons, 2006; Büssing et al., 2012b; Domingues, 2018). Some of the reviewed studies reported positive associations of spirituality and psychological health outcomes, specifically among yoga practitioners. Spirituality (more precisely, spiritual readiness) was positively associated with body awareness (*r* = 0.29, *p* < 0.01), body responsiveness (*r* = 0.31, *p* < 0.01), and body satisfaction (*r* = 0.297, *p* < 0.01) (Dittmann and Freedman, 2009). Moreover, spiritual well-being was negatively associated with depression (*r* = –0.47, *p* < 0.01), anxiety (*r* = –0.28, *p* < 0.01) and stress (*r* = -0.54, *p* < 0.01) (Nandeesh et al., 2016). Although these two studies are the only ones included in this review, which investigated such associations, their results may support the idea that spirituality could be a strategy to increase well-being (Henry, 2006). In order to increase spirituality, a regular and in-depth practice is fundamental. Incorporation of yoga philosophy, meditation, consecration, prayer, as well as the support of the community (teachers and fellow practitioners), is essential for spiritual growth (Ness and Briles, 2016; Pandya, 2018).

We can assume that yoga practice can affect psychological health in at least two ways: either directly or through enhancing spirituality. However, further specific studies are needed to explore the relationship of yoga, spirituality, and mental health (MacDonald, 2013).

### Spirituality Questionnaires

The analyzed studies used various questionnaires to measure spirituality. These could be roughly categorized into four groups: questionnaires measuring (1) spirituality among diseased populations, (2) spiritual experience (s), (3) thinking style or a worldview, and (4) spirituality as a construct, including various (sometimes a myriad of) facets. Two measures were designed for diseased populations, namely the functional assessment of chronic illness therapy were used—spiritual well-being scale (FACIT-Sp) (Peterman et al., 2002) and the spiritual attitudes and coping with illness (SpREUK) scale (Büssing et al., 2005). Spiritual experiences were assessed as self-transcendence, spiritual connection, gratitude, or hope. The respective measures were, e.g., the daily spiritual experiences scale (Underwood and Teresi, 2002) and the spiritual connection questionnaire (SCQ-14) (Wheeler and Hyland, 2008). Spiritual thinking style or a worldview was measured, using, for instance, the scale for existential thinking (SET) (Allan and Shearer, 2012) and the worldview scale (De Witt, 2013). Furthermore, several studies applied questionnaires to measure spirituality as a complex construct, such as the spiritual intelligence self-report inventory (SISRI) (King and DeCicco, 2009), the aspects of spirituality (ASP) scale (Büssing et al., 2010), or the multidimensional inventory for religious/spiritual well-being (MI-RSWB) (Unterrainer et al., 2012). For the full list of spirituality questionnaires used by the analyzed studies, see the Appendix 3. Overall, the divergence of the questionnaires and their underlying concepts is obvious. There are a variety of instruments but a lack of consensus on how to best assess spirituality (MacDonald, 2013). The results of this review are, nonetheless, by and large, positive. The applied measures captured predominantly positive outcomes. Even so, in future research, it is important to consider appropriate measures of spirituality in yoga. A wide range of instruments seems to be promising, and recommendations are available (MacDonald and Friedman, 2009; MacDonald, 2013).

## Discussion

According to the quantitative and qualitative findings, although most of the studies showed risk of bias, it can be concluded that yoga practice may be positively associated with spirituality. While this association is most likely dependent on the exact nature of the practice and the background, intention, and preexisting relationship of the practitioner with spirituality, yoga interventions appear to have the potential to enhance different aspects of spirituality. These aspects include spiritual aspirations, a search for insight/wisdom, existential thinking, a sense of meaning and peace, as well as the feeling of faith, hope, and compassion. In summary, yoga practice may improve various aspects of spiritual well-being and spiritual intelligence. Yoga practice may also be associated with increased levels of spiritual health, a more positive outlook on life, happiness within, and lower levels of existential anxieties. Accordingly, yoga can be a method that facilitates spiritual well-being and health, spiritual growth, and the development of an integrative worldview. To achieve these benefits, regular yoga practice is essential. Consequently, the practice itself is a key component for spiritual growth. Concerning the intention to practice, physical and appearance motivations seem to be more prevalent than spiritual ones, which holds for both beginner and advanced Western practitioners. Even so, spiritual intentions may increase over time and with a more in-depth practice, which suggests that hatha yoga can cultivate spirituality. According to the studies included in this review, for yoga practitioners, spirituality provides a meaning and a framework of life and is a way of gaining more self-awareness and improving oneself. Incorporation of yoga philosophy, meditation, consecration, and prayer may foster experiences of inner peace, freedom, and connectedness.

### Outcomes of Recent Studies

For further evidence of the investigated topic, we also inspected the most recent articles, which meet the inclusion criteria of the present review. For this purpose, we conducted the same literature search as registered in Prospero in 2019 for the time interval between the end of 2019 and 2021 (May/June). This second search resulted in three new articles (Park et al., 2020; Bringmann et al., 2021; Bryan et al., 2021). We, moreover, identified three additional recent papers (Cartwright et al., 2020; Csala et al., 2020; Büssing et al., 2021). Summaries of these articles are presented in Table 2. All six studies used a quantitative approach, two of them had a cross-sectional design, and four papers applied an intervention (one with a control group). All of them were conducted in Western countries (either Europe or the United States). The results of the six recent articles are, in general, in accordance with the outcomes of this review and support its findings. In addition, they provide further insights into the relationship between yoga and spirituality. The results of two studies investigating diseased populations (Bringmann et al., 2021; Bryan et al., 2021) are in line with previous inconclusive outcomes. Patients with a current diagnosis of a mild or moderate depressive episode showed a higher level of a search for insight/wisdom, but no changes in the other three measured aspects of spirituality (religious orientation, conscious interactions, and transcendence conviction) after an 8-week-long complex intervention (Bringmann et al., 2021). Cancer survivors reported increased overall spiritual well-being after a 10-week-long gentle yoga and mindfulness meditation program (Bryan et al., 2021). Concerning the motivations to practice, one study (Cartwright et al., 2020) supported that initial reasons to practice are mostly connected to physicality and health (that is, general wellness, fitness, and flexibility), while the most notable change in motivations over time concerns spirituality. Initially, 2.5% of the participants reported to have spiritual motivations, whereas this number rose to 21%. Still the highest current motivation was general well-being with 28%. Among regular yoga practitioners (with an average of 13 years of yoga practice), 78% considered yoga a spiritual path, while only 26% considered it gymnastics (Büssing et al., 2021). The participants perceiving yoga as a spiritual path show higher levels of devotion/self-reflection/contentment, restrain/truthfulness/self-discipline, and non-possessiveness (measured by the Yama/Niyama Questionnaire) compared with those who see yoga as gymnastics (Büssing et al., 2021). A 10-week-long intervention study (Csala et al., 2020) investigated the role of verbal instruction during physically identical hatha yoga practice among beginner students compared with no-intervention controls. One yoga group received cuing, which focused on physical aspects of practice, while, in the other yoga group, it included philosophical and spiritual contents (the amount of instruction was equal). No differences between the three groups emerged; however, the two yoga groups merged showed an increased level of spiritual connection compared with controls at the end of the intervention. This indicates that yoga practice can enhance spiritual connection regardless of verbal information. Another study (Park et al., 2020) examined the acute effects of various types of hatha yoga on psychological resources and emotional well-being among regular yoga practitioners (with an average of 13 years of yoga practice). Increased levels of self-transcendence and spiritual peace were reported after a 60-min-long yoga class independently of style. Both changes in self-transcendence and spiritual peace favorably correlated with changes in emotions. Additionally, warmth and friendliness of the yoga teacher positively correlated with self-transcendence. According to these two studies (Csala et al., 2020; Park et al., 2020), even exercise-based hatha yoga practice may be able to evoke aspects of spirituality, both for beginners and regular practitioners. Attitude or characteristics of the yoga teacher may also play a role in this process. Summarizing the outcomes of the systematic review and the recent findings, it can be said that motivations to practice yoga may change over time; initial physical intentions may gradually be complemented by spiritual ones. A potential explanation might be that the practice of hatha yoga as a form of exercise may trigger the experience of aspects of spirituality. In doing so, it may evoke interest in aspects of yoga, which go beyond mere exercise (e.g., meditation and philosophical studies), which, in turn, can enhance spirituality.

**TABLE 2:** Summary of the articles between 2020 and 2021 (all with quantitative approach).

Article	Design	Sample size (yoga group)	Control group(s)	Sample condition, age, gender	Measurement tool	Type of yoga practice	Frequency of intervention/previous experience with yoga	Major findings	Origin of the study
Bringmann et al., 2021	Longitudinal	*N* = 20	No	Patients with a current diagnosis of a mild or moderate depressive episode; mean age: NR; 88% female	Aspects of Spirituality (ASP; Büssing et al., 2010), four subscales: (1) Religious orientation: prayer/trust in God, (2) Search for insight/wisdom, (3) Conscious interactions/compassion, (4) Transcendence conviction	Meditation Based Lifestyle Modification (MBLM) - a complex mind-body intervention: P, B, R, M LS, RB, L (yama, niyama emphasized), Ma	8 weeks, weekly 3-h class + 45 min daily home practice recommended	Increase in Search for Insight/wisdom, but no changes in the other 3 subscales at the end of the intervention	Europe (Germany)
Bryan et al., 2021	Longitudinal	*N* = 8	No	Cancer survivors; mean age: 61 years; 87.5% female	FACIT-Sp (Peterman et al., 2002), three subdomains: meaning, peace, and faith; two open-ended questions	Gentle yoga and mindfulness meditation: P, B, R, M	10 weeks; 45 min classes two times/week	Increased spiritual well-being at the end of the intervention; increased levels of meaning and faith, but no changes in peace; reported spiritual perceptions: peaceful, tranquil, opportunity for prayer, spiritually connected	USA
Büssing et al., 2021	Cross-sectional	*N* = 901	No	Regular yoga practitioners; mean age: 49 years; 89% female	Interpretation of yoga; Yama/Niyama Questionnaire (YaNiQ; Büssing et al., 2021)	Various forms of yoga: 88% Yoga Vidya	Average number of years of yoga practice: 13, Average amount of yoga practice 55% several times/week; 31% daily; 11% once/week, 3% less than once/week	Yoga as a spiritual path for 78%; Participants’ perspectives on their practice whether it is seen as gymnastics or spiritual path influence yama/niyama scores: participants regarding yoga as a spiritual path have higher scores for (1) Devotion/Self-Reflection/Contentment, (2) Restrain/Truthfulness/Self-Discipline, and (3) Non-Possessiveness, but not for (4) Non-Harming	Europe (Germany)
Cartwright et al., 2020	Cross-sectional	*N* = 2434	No	Adult United Kingdom residents practicing yoga within the previous 12 months; mean age: 48.7 years; 87.3% female	Motivation for yoga practice	Various forms of hatha yoga	Average number of years of yoga practice: 13.9; Average amount of yoga practice: 5.52 h/week	Initial reasons were general wellness (39%), fitness (19%) and flexibility (8.5%); most notable change in spirituality over time: recent spiritual motivation 21%	Europe (United Kingdom)
Csala et al., 2020	Longitudinal	*N* = 50, two subgroups: Sport group (physical aspect emphasized): *N* = 27, Spiritual group (philosophical and spiritual contents included): *N* = 23	*N* = 34, no intervention	Beginner female students; mean age: 22 years, 100% female	Spiritual Connection Questionnaire (SCQ-14; Wheeler and Hyland, 2008)	Beginner hatha yoga; Sport group: P, B, R; Spiritual group: P, B, R, Phi, Ma	10 weeks; weekly 1.5 h class, no home practice	No differences between the two yoga groups; Increased spiritual connection in the merged yoga group compared to control at the end of the intervention	Europe (Hungary)
Park et al., 2020	Interventional	*N* = 144	No	Regular yoga practitioners (average number of years of yoga practice: 7.1); mean age: 40 years; 78% female	Psychological resources: Self-Transcendence Subscale of the Temperament and Character Inventory (STS; MacDonald and Holland, 2002); Peace subscale of FACIT-Sp (Canada et al., 2008)	11 forms of hatha yoga: included components measured by Essential Properties of Yoga Questionnaire (EPYQ; Park et al., 2018)	1-h long single yoga session	Increased self-transcendence and spiritual peace after yoga class independently of style; both changes in self-transcendence and spiritual peace favorably correlated with changes in emotions; higher levels of warmth and friendliness of the yoga teacher correlate with increased self-transcendence	United States

*NR, not reported; P, posture; B, breathing exercise; R, relaxation; M, meditation; LS, lifestyle advice; RB, reading books, manuals, or other teachings; Phi, philosophy, L, lectures on philosophy; Ma, mantras; FACIT-Sp, Functional Assessment of Chronic Illness Therapy – Spiritual Well-Being Scale.*

So far, only a few studies have investigated the relationship between yoga and spirituality, with holistic yoga practices being a particularly poorly studied area. In line with previous authors (Büssing et al., 2012a; MacDonald, 2013), we suggest further exploration of this topic. First of all, aspects of spirituality most strongly associated with yoga practice could be revealed. For that, complex questionnaires or simultaneous application of more one-facet measures assessing spirituality in a non-religious form are suggested. For measurement of yoga experience, the yoga immersion scale (YI-S) (Gaiswinkler and Unterrainer, 2016) and for measurement of types and components of yoga practice The essential properties of yoga questionnaire (EPYQ) (Park et al., 2018) are, for instance, recommended. Secondly, potential diverse effects or different levels of impact of exercise-based or holistic yoga programs need future investigation. In addition, we propose a comparison of Eastern and Western approaches to yoga. Underlying mechanisms of the positive effects of yoga practice on spirituality also require deeper understanding. For that, intervention studies with randomized control design are necessary to avoid potential biases. Lastly, it is worthwhile to explore the intercorrelation of spirituality and other positive mental health measures affected by yoga.

### Limitations

The present systematic review has a few limitations. First of all, it does not discuss how spirituality and religiosity relate to each other. Thus, the search terms were narrowed down to “spirituality,” “spiritual,” and “spirit.” Consequently, studies using the terms “religiosity” or “religious” but capturing aspects of spirituality are not included in this review. Similarly, other overlapping terms, such as “transcendence,” “transcendental,” “self-transcendence,” “mystic,” “mystical,” “mysticism,” “sacred,” “divine,” or “philosophical” were excluded from the literature search as well. Secondly, the present review is restricted to empirical studies published in peer-reviewed scientific journals. Theses, dissertations, and other writings were not eligible, limiting the number of included papers profoundly. For a methodologically sound analysis, however, the strict delimitations were necessary. The aim of the restriction was to gain an overview and synthesize the outcomes of the presumably most robust and reliable studies in this field. Nevertheless, methodologies (including approach, design, sample, intervention, and measures) of the analyzed studies are heterogeneous, and it is not possible to draw statistical evidence of the topic, which presents a further limitation of the review. Also, the majority of the studies did not include a control group, which makes it difficult to determine whether similar outcomes could have been achieved through other practices. Risks of bias assessment of RCTs and cross-sectional studies also showed some high risk of bias. This suggests the cautious interpretation of the outcomes and further exploration of the topic with proper randomization processes, preregistered study design, and proper data analysis. Furthermore, most of the studies did not report on the motivations of the participants to practice yoga, which is another important potential bias of the results. We do not know whether attendants are prone to or interested in spirituality and whether this is why yoga practice facilitates spiritual expression for them, or in turn, whether yoga kindles and enhances spirituality for people who are originally not open to it. Of course, these options are not mutually exclusive. Lastly, it is important to note that some of the measured aspects of spirituality (e.g., hope, conscious interaction, an integrative worldview) are not “spiritual” *per se*. They could also be understood as separate concepts or even as outcomes of spirituality. Even so, the present review aimed to explore which aspects of spirituality are, in fact, measured in yoga research and not to determine or delimit the definition of spirituality. Similarly, terms such as “spiritual well-being” or “spiritual health” and their respective measures share aspects with general mental health and should, therefore, be interpreted cautiously. However, they do encompass aspects that go beyond general mental health or well-being (Vader, 2006; Bredle et al., 2011; Unterrainer et al., 2012).

## Conclusion

Based upon the 30 reviewed studies and six additional recent studies, yoga practice seems to be positively associated with various aspects of spirituality and may be a valid method to evoke spiritual interest and foster spiritual well-being and health. For most benefits, regular yoga practice is essential. Even though physical fitness appears to be the most important aspect of yoga practice in Western society, spiritual benefits are still manifested. For more evidence and a deeper exploration of the specific effects of yoga on spirituality, future research is encouraged.

## Data Availability Statement

The original contributions presented in the study are included in the article/Supplementary Material, further inquiries can be directed to the corresponding author/s.

## Author Contributions

All the authors contributed to the conception and the design of the review, and read, commented, and approved the last version of the manuscript. BC and CMS conducted the literature search, study selection, data extraction, and synthesis of the outcomes. FK resolved the disagreements between BC and CMS in the selection phase and any further problematic issues. BC wrote the first draft of the manuscript. CMS made major improvements and edited the language.

## Conflict of Interest

The authors declare that the research was conducted in the absence of any commercial or financial relationships that could be construed as a potential conflict of interest.

## Publisher’s Note

All claims expressed in this article are solely those of the authors and do not necessarily represent those of their affiliated organizations, or those of the publisher, the editors and the reviewers. Any product that may be evaluated in this article, or claim that may be made by its manufacturer, is not guaranteed or endorsed by the publisher.

## Supplementary Material

Supplementary Table 1 Summary of the articles with quantitative approach.
