# Supplementary material for: The Relationship Between Yoga and Spirituality: A Systematic Review of Empirical Research
Source: Front Psychol. 2021 Aug 2;12:695939. doi: 10.3389/fpsyg.2021.695939 (PMC8365182; doi:10.3389/fpsyg.2021.695939)
Supplement: Supplementary Table 1 — Summary of the articles with quantitative approach. [file Data_Sheet_1.pdf]

Appendices

Appendix 1

Evaluation of the risk of bias (in 5 domains and overall) in RCT studies using RoB 2.0 tool (Higgins et al., 2011)

**Article**	**Randomization process**	**Deviations from intended intervention**	**Missing Outcome data**	**Measurement of the outcome**	**Selection of reported result**	**Overall**
Danhauer et al., 2009	Low	Low	Low	Some	Some	Some
Lötzke et al., 2016	Low	Low	Low	Some	Some	Some
Moadel et al., 2007	Some	Low	High	Some	Some	High
Pandya, 2019	High	Low	Some	Some	Some	High
Safara & Ghasemi, 2017	Low	Low	Some	Some	Some	Some
Smith et al., 2011	Some	Low	High	Some	Some	High

Note. Low: Low concern, Some: Some concern, High: High concern.

Appendix 2

Evaluation of the risk of bias of cross-sectional studies with quantitative approach using JBI critical appraisal checklist for analytical cross-sectional studies (Moola et al., 2020)

**Article**	1.Were the criteria for inclusion in the sample clearly defined?	2. Were the study subjects and the setting described in detail?	3. Was the exposure measured in a valid and reliable way?	4. Were objective, standard criteria used for measurement of the condition?	5. Were confounding factors identified?	6. Were strategies to deal with confounding factors stated?	7. Were the outcomes measured in a valid and reliable way?	8. Was appropriate statistical analysis used?	**Yes %**	**No %**
Ahmad & Imtiaz, 2016	N	N	N	Y	N	N	Y	Y	37.5	62.5
Csala et al., 2017	N	Y	Y	Y	Y	Y	Y	Y	87.5	12.5
Dittmann & Freedman, 2009	Y	Y	Y	Y	N	N	Y	Y	75	25
Gaiswinkler & Unterrainer, 2016	Y	Y	Y	Y	Y	Y	Y	Y	100	0
Ivtzan & Jegatheeswaran, 2015	Y	Y	Y	Y	N	N	Y	Y	75	25
Ivtzan & Papantoniou, 2014	Y	N	Y	Y	N	N	Y	Y	62.5	37.5
Moliver et al., 2013	Y	Y	Y	Y	Y	Y	Y	Y	100	0
Monk-Turner & Turner, 2010	N	N	N	Y	N	N	Y	Y	37.5	62.5
Nandeesh et al., 2016	Y	N	N	Y	N	N	Y	Y	50	50
Park et al., 2016	Y	Y	Y	Y	N	N	Y	Y	75	25
Seena et al., 2017	Y	N	N	Y	N	N	Y	Y	50	50

Quilty et al., 2013	Y	Y	Y	Y	N	N	Y	Y	75	25

Note. Y: Yes, N: No. (No results of "Unclear" or "Not applicable").

Appendix 3

List of spirituality questionnaires used by the analyzed studies
(numbers indicate the number of studies which include the given questionnaire)

- Functional Assessment of Chronic Illness Therapy - Spiritual Well-Being Scale (FACIT-Sp) (Peterman et al., 2002) (6)
- Spiritual Attitudes and Coping with Illness (SpREUK) (Büssing et al., 2005) (1)
- Religious Commitment Inventory-10 (RCI-10) (Worthington et al., 2003) (1)
- Spiritual Intelligence Self-Report Inventory (SISRI) (King & DeCicco, 2009) (2)
- Self-Transcendence Survey (Reed, 1991) (1)
- Spiritual Connection Questionnaire (SCQ-14) (Wheeler & Hyland, 2008) (1)
- Daily Spiritual Experiences Scale (DSES) (Underwood & Teresi, 2002) (2)
- Gratitude Questionnaire-Six Item Form (GQ-6) (McCullough et al., 2002) (1)
- Herth Hope Scale (HHS) (Herth, 1992) (1)
- Aspirations Index (AI) (Kasser & Ryan, 1996) (1)
- Scale for Existential Thinking (SET) (Allan & Shearer, 2012) (1)
- Existential Anxiety Questionnaire (EAQ) (Weems et al., 2004) (1)
- Worldview Scale (WS) (De Witt, 2013) (1)
- Meaning in Life Questionnaire (MLQ) (Steger et al., 2006) (1)
- Spiritual Readiness Scale (Dittmann & Freedman, 2009) (1)
- Spirituality Scale (SS) (Hardt et al., 2012) (1)
- Aspects of Spirituality (ASP) (Büssing et al., 2010) (1)
- Spirituality Index of Wellbeing Scale (SIWS) (Daaleman & Frey, 2004) (1)
- Body, Mind, Spirit Wellness and Characteristic Inventory (BMS-WBCI) (Hey et al., 2006) (1)
- Bio-Psycho-Social-Spiritual Scale (BPSS) no citation (1)
- Multidimensional Inventory for Religious/Spiritual Well-Being (MI-RSWB) (Unterrainer et al., 2012) (1)
- The Subjective Well-Being Inventory- Transcendence subscale (SUBI-T) (Sell, 1994) (1)

References

Allan, B., & Shearer, B. (2012). The Scale for Existential Thinking. *International Journal of Transpersonal Studies, 31*, 21–37. https://doi.org/10.24972/ijts.2012.31.1.21

Büssing, A., Föller-Mancini, A., Gidley, J., & Heusser, P. (2010). Aspects of spirituality in adolescents. *International Journal of Children's Spirituality*, *15*(1), 25–44. https://doi.org/10.1080/13644360903565524

Büssing, A., Ostermann, T., & Matthiessen, P. F. (2005). Role of religion and spirituality in medical patients: Confirmatory results with the SpREUK questionnaire. *Health and Quality of Life Outcomes, 3*(1), 10. https://doi.org/10.1186/1477-7525-3-10

Daaleman, T. P., & Frey, B. B. (2004). The Spirituality Index of Well-Being: A New Instrument for Health-Related Quality-of-Life Research. *Annals of Family Medicine*, *2*(5), 499–503. https://doi.org/10.1370/afm.89

De Witt, A. (2013). Worldviews and Their Significance for the Global Sustainable Development Debate. *Environmental Ethics*, *35*, 133–162. https://doi.org/10.5840/enviroethics201335215

Dittmann, K. A., & Freedman, M. R. (2009). Body Awareness, Eating Attitudes, and Spiritual Beliefs of Women Practicing Yoga. *Eating Disorders, 17*(4), 273–292. https://doi.org/10.1080/10640260902991111

Hardt, J., Schultz, S., Xander, C., Becker, G., & Dragan, M. (2012). The Spirituality Questionnaire: Core Dimensions of Spirituality. *Psychology, 3*, 116–122. https://doi.org/10.4236/psych.2012.31017

Herth, K. (1992). Abbreviated instrument to measure hope: Development and psychometric evaluation. *Journal of Advanced Nursing, 17*(10), 1251–1259. https://doi.org/10.1111/j.1365-2648.1992.tb01843.x

Hey, W. T., Calderon, K. S., & Carroll, H. (2006). Use of body-mind-spirit dimensions for the development of a wellness behavior and characteristic inventory for college students. *Health Promotion Practice*, *7*(1), 125–133. https://doi.org/10.1177/1524839904268525

Kasser, T., & Ryan, R. (1996). *Further Examining the American Dream: Differential Correlates of Intrinsic and Extrinsic Goals*. https://doi.org/10.1177/0146167296223006

King, D., & DeCicco, T. (2009). A Viable Model and Self-Report Measure of Spiritual Intelligence. *International Journal of Transpersonal Studies, 28*, 68–85. https://doi.org/10.24972/ijts.2009.28.1.68

McCullough, M. E., Emmons, R. A., & Tsang, J.-A. (2002). The grateful disposition: A conceptual and empirical topography. *Journal of Personality and Social Psychology*, *82*(1), 112–127. https://doi.org/10.1037/0022-3514.82.1.112

Peterman, A. H., Fitchett, G., Brady, M. J., Hernandez, L., & Cella, D. (2002). Measuring spiritual well-being in people with cancer: The functional assessment of chronic illness therapy--Spiritual Well-being Scale (FACIT-Sp). *Annals of Behavioral Medicine: A Publication of the Society of Behavioral Medicine*, *24*(1), 49–58. https://doi.org/10.1207/S15324796ABM2401_06

Reed, P. G. (1991). Self-transcendence and mental health in oldest-old adults. *Nursing Research*, *40*(1), 5–11.

Sell, H. (1994). The Subjective Well-Being Inventory (SUBI). *International Journal of Mental Health*, *23*(3), 89–102. https://doi.org/10.1080/00207411.1994.11449289

Steger, M. F., Frazier, P., Oishi, S., & Kaler, M. (2006). The meaning in life questionnaire: Assessing the presence of and search for meaning in life. *Journal of Counseling Psychology, 53*(1), 80–93. https://doi.org/10.1037/0022-0167.53.1.80

Underwood, L. G., & Teresi, J. A. (2002). The Daily Spiritual Experience Scale: Development, theoretical description, reliability, exploratory factor analysis, and preliminary construct validity using health-related data. *Annals of Behavioral Medicine*, *24*(1), 22–33. https://doi.org/10.1207/S15324796ABM2401_04

Unterrainer, H.-F., Nelson, O., Mcgrath, J., & Fink, A. (2012). The English Version of the Multidimensional Inventory for Religious/Spiritual Well-Being (MI-RSWB-E): First Results from British College Students. *Religions, 3*, 588–599. https://doi.org/10.3390/rel3030588

Weems, C. F., Costa, N. M., Dehon, C., & Berman, S. L. (2004). Paul Tillich's theory of existential anxiety: A preliminary conceptual and empirical examination. *Anxiety, Stress & Coping: An International Journal, 17*(4), 383–399. https://doi.org/10.1080/10615800412331318616

Wheeler, P., & Hyland, M. E. (2008). The development of a scale to measure the experience of spiritual connection and the correlation between this experience and values. *Spirituality and Health International*, *9*(4), 193–217. https://doi.org/10.1002/shi.348

Worthington, E., Wade, N., Hight, T., Ripley, J., Mccullough, M., Berry, J., Schmitt, M., Berry, J., Bursley, K., & O'Connor, L. (2003). The Religious Commitment Inventory—10:

Development, Refinement, and Validation of a Brief Scale for Research and Counseling. *Journal of Counseling Psychology*, *50*, 84–96. https://doi.org/10.1037/0022-0167.50.1.84
