# Supplementary material for: The Relationship Between Yoga and Spirituality: A Systematic Review of Empirical Research
Source: Front Psychol. 2021 Aug 2;12:695939. doi: 10.3389/fpsyg.2021.695939 (PMC8365182; doi:10.3389/fpsyg.2021.695939)
Supplement: Supplementary file 2 [file Table_1.pdf]

Tables

Table 1
Summary of the articles with quantitative approach

**Article**	**Design**	**Sample size (yoga group)**	**Control group(s)**	**Sample condition, age, gender**	**Measurement tool**	**Type of yoga practice**	**Frequency of intervention/previous experience with yoga**	**Major findings**	**Origin of the study**
Ahmad & Imtiaz, 2016	cross-sectional	N=60	no	Participants from various Yoga Training Centers and Institutions of Delhi and NCR; mean age: NR, age between 35-60 yrs; gender: NR	Spirituality Scale (SS; Hardt et al., 2012), 4 subscales: 1. Belief in God, 2. Search for meaning, 3. Mindfulness, 4. Feeling of security	NR	NR	Among yoga practitioners, spirituality is not associated with avoidant and anxious attachment to God	India
Baker et al., 2015	longitudinal	N=49	no	Veterans with severe mental illness, with various previous experience of yoga; mean age: 61.4 yrs; 9.2% female	Bio Psycho-Social-Spiritual Scale (BPSS; no citation)	Yoga-based wellness classes: "Breathing, Stretching, Relaxation" (BSR) P, B, R	8 weeks; 3 different groups: short (< 30 min), medium (30-59 min), and long (> 60 min) classes	"Perceived benefits of yoga" is associated with spiritual well-being, class duration, duration of attendance, and frequency of attendance	USA
Büssing et al., 2012	longitudinal	N=160	no	Participants starting a 2-year yoga teacher training in Yoga Vidya Centers; mean age: 40.9 yrs; 91% female	Aspects of Spirituality (ASP; Büssing et al., 2010), 4 subscales: 1. Religious orientation: prayer/trust in God, 2. Search for insight/wisdom, 3. Conscious interactions/compassion, 4. Transcendence conviction	Intensive yoga practice: P, B, R, M, Ma, LS, RB, L	6 months; weekly 3-hour evening tutorial in small groups + intensive training weekends about every 2 months + 1 hour/day home practice	Increase in Conscious interactions/compassion, and Religious orientation: prayer/trust in God; marginal increase in Search for Insight/wisdom; no change in Transcendence conviction	Europe (Germany)
Csala et al., 2017	cross-sectional	N=85	no	Regular yoga practitioners;	Spiritual Connection Questionnaire (SCQ-	NR (postural yoga)	Average number of years of yoga practice 7.7;	Spirituality is associated with	Europe (Hungary)

				mean age: 43.8 yrs; 84.7% female	14; Wheeler and Hyland, 2008)		Average amount of yoga practice: 4.9 hours/week	regularity of yoga practice (hours/week)	
Danhauer et al., 2008	longitudinal	N=51	no	Women with ovarian or breast cancer (any stage; 61% actively undergoing cancer treatment); mean age: 58.9 yrs; 100% female	FACIT-Sp (Peterman et al., 2002), 2 subscales: 1. Sense of meaning/peace, 2. Role of faith	Restorative yoga: P, B, R + ahimsa emphasized	10 weeks; weekly 75 min class	No changes in spirituality	USA
Danhauer et al., 2009*	longitudinal	N=22	N=22, waitlist control	Women with breast cancer (any stage; 34% actively undergoing cancer treatment); mean age: 55.8 yrs; 100% female	FACIT-Sp (Peterman et al., 2002), 2 subscales: 1. Sense of meaning/peace, 2. Role of faith	Restorative yoga: P, B, R + ahimsa emphasized	10 weeks; weekly 75 min class	Improvement of Sense of meaning/peace in the yoga group	USA
Dittmann & Freedman, 2009; Study 1.	cross-sectional	N=129, 2 subgroups: G1: who practiced primarily for psychospiritual reasons N=99, G2: who practiced primarily for physical or appearance reasons N=30	no	Regular yoga practitioners in yoga and fitness centers; mean age: 47.4 yrs; 100% female	Spiritual Readiness Scale (established by the authors), 2 subscales: 1. Meaningfulness of ritual, 2. Importance of seeking	NR (postural yoga)	Average number of years of yoga practice: 12.1; at least 1 hour/week	Spirituality is higher in women practicing with psychospiritual reasons; spirituality is associated with Body Awareness, Body Responsiveness, Intuitive Eating, and Body Satisfaction	USA
Gaiswinkler & Unterrainer, 2016	cross-sectional	N=362, 3 subgroups according to Yoga Immersion Scale (YI-S):	N=93, gymnastics (mixed methods**)**	Regular yoga practitioners in yoga and fitness studios; mean age: 33.53 yrs; 90.3% female	Multidimensional Inventory for Religious/Spiritual Well-Being (MI-RSWB; Unterrainer et al., 2012),	Postural yoga: Hatha Yoga, Bikram Yoga, Ashtanga	Involvement with yoga is assessed by the Yoga Immersion Scale (YI-S)	Yoga immersion, i.e., duration and frequency, is associated with Religious/Spiritual Well-Being;	Europe (Austria)

		Y1: Yoga marginal N=111, Y2: Yoga moderate N=120, Y3: Yoga high N=131			6 subscales: 1. General religiosity, 2. Forgiveness, 3. Connectedness, 4. Hope immanent, 5. Hope transcendent, 6. Experiences of sense and meaning	Yoga, Iyengar Yoga, Karma Yoga, Hormone Yoga		Religious/Spiritual Well-Being is higher in practitioners with more practice	
Ivtzan & Jegatheeswaran, 2015	cross-sectional	N=235	no	Regular yoga practitioners; mean age: 40.25 yrs; 81.7% female	Motivation for yoga practice at beginning and continued: comparing physical fitness and spiritual motives	Various forms of hatha yoga (postural yoga)	Diverse range of yoga experience: Number of years of yoga practice: 11% for less than 1 year, 55% for up to 10 years, 23% for 10-20 years; Amount of yoga practice: 31% daily, 35% at least 3 times/week, 34% at least 1 time/week	Greater initial and continued physical intentions than spiritual intentions; spiritual intentions become more salient over time suggesting that Western yoga can cultivate spirituality; practitioners with spiritual intentions report higher psychological well-being	Europe (UK)
Ivtzan & Papantoniou, 2014	cross-sectional	N=124	no	Regular yoga practitioners and participants who had no experience with yoga; mean age: 40.65 yrs; 72.5% female	Meaning in Life Questionnaire (MLQ; Steger et al., 2006); Gratitude Questionnaire-Six Item Form (GQ-6) (McCullough et al., 2002)	Various forms of hatha yoga	82 regular practitioners: Number of years of yoga practice: 39% up to 1 year, 23% for 2 years, 38% more than 2 years; Amount of yoga practice: at least 2 times/week; 42 participants with no experience with yoga	Experience with yoga (number of years practicing yoga) is associated with Meaning in life and Gratitude	Europe (UK)

Lötzke et al., 2016*	longitudinal	N=45	N=47, conventional physical exercise	Women with breast cancer (stages I-III; 100% undergoing oncological treatment) (no previous experience with yoga); mean age: 51 yrs; 100% female	Spiritual Attitudes and Coping with Illness (SpREUK; Büssing et a., 2005), 3 subscales: 1. Search for support/access to spirituality/religiosity, 2. Trust in higher guidance/source, 3. Reflection: positive interpretation of disease	Regenerative Iyengar yoga: P, B	12 weeks; weekly 1 hour + 2 times/week 20 min home practice (guided by written instructions)	Increase of Reflection: positive interpretation of disease;<br>decrease to initial level after 3 months; no differences between yoga and control group	Europe (Germany)
Moadel et al., 2007*	longitudinal	N=84	N=44, waitlist control	Women with breast cancer (stages I to III; 48% receiving oncological treatment: chemotherapy or antiestrogen therapy); mean age: 52.1 yrs; 100% female	FACIT-Sp (Peterman et al., 2002), 2 subscales: 1. Sense of meaning/peace, 2. Role of faith	Based on hatha yoga: P, B, M	12 weeks; weekly 1.5 hours class	No change in spirituality;<br>among patients not receiving chemotherapy: deterioration of spirituality in control compared to yoga group	USA
Moliver et al., 2013	cross-sectional	N=211		Regular yoga practitioners over 45 years; mean age: NR; 100% female	The Subjective Well-Being Inventory - Transcendence subscale (SUBI-T; Sell, 1994)	Hatha yoga: P, B, R, M	Average number of years of yoga practice: NR; Average amount of yoga practice: 4.21 hours/week and 2.56 hours/week of seated meditation	Transcendence is most strongly associated with frequency of current yoga practice, furthermore with total lifetime hours and total calendar years spent in yoga	USA
Monk-Turner & Turner, 2010	cross-sectional	N=61	N=126, non-yoga practitioners	Regular yoga practitioners over 18 yrs; mean age: NR; 62% female in the total sample, 77%	Body, Mind, Spirit Wellness and Characteristic Inventory (BMS-WBCI; Hey et al., 2006)	Hatha yoga	Regular practitioners, further details NR	Group differences in 5 out of 9 items: yoga practitioners are more likely to (1) express their spirituality appropriately and in healthy ways, (2)	USA

				female in the yoga group				recognize the positive contribution faith could make to the quality of life, (3) routinely undertake new experiences to enhance spiritual health, (4) have a positive outlook on life, and (5) experience happiness within	
Nakau et al., 2013	longitudinal	N=22	no	Breast and lung cancer patients (varied stages; 0% actively undergoing cancer treatment); mean age: 58.1 yrs; 82% female	FACIT-Sp (Peterman et al., 2002), 2 subscales: 1. Sense of meaning/peace, 2. Role of faith	Yoga practice: P, B, R, M (as a part of integrated therapy)	12 weeks; weekly 1.5 hours yoga session + daily video guided home practice	Increased spiritual well-being at the end of the intervention	Japan
Nandeesh et al., 2016	cross-sectional	N=80	no	Regular yoga practitioners; mean age: 40.5 yrs; 50% female	FACIT-Sp (Peterman et al., 2002), 2 subscales: 1. Sense of meaning/peace, 2. Role of faith	NR	Number of years of yoga practice: at least one year	Among yoga practitioners, spirituality shows negative correlation with depression, anxiety and stress	India
Pandya, 2017	longitudinal	N=1914	no	Participants of Raja Yoga spiritual program of the Brahmakumaris across nine countries; mean	Spirituality Index of Wellbeing Scale (SIWS; Daaleman & Frey, 2004)	Raja yoga: reading of literature, listening to talks/lectures, watching meditation	One year; Raja Yoga courses on a regular basis	Increased spiritual well-being after program participation	India

				age: NR, over 20 yrs; 53.3% female		videos, and self-practice M, LS, RB, L, Ma			
Pandya, 2018; Part 1.	longitudinal	N=744	no	Auroville spiritual community residents; Post intervention mean age: 42.7 yrs; 70% female	Scale for existential thinking (SET; Allan & Shearer, 2012); Aspirations Index (AI; Kasser & Ryan, 1996), 2 subscales: 1. Extrinsic aspirations of wealth, fame and image, 2. Intrinsic aspirations of meaningful relationships; Existential Anxiety Questionnaire (EAQ; Weems et al., 2004); Daily Spiritual Experiences Scale (DSES; Underwood & Teresi, 2002); Worldview Scale (WS; De Witt, 2013)	Integral yoga of Sri Aurobindo LS	10 years; residence in Auroville spiritual community	Increased levels of existential thinking, aspirations, spiritual experiences and integrative worldview, and decreased level of existential anxieties after 10 years of residence	India
Pandya, 2019*	longitudinal	N=3488	N=3488, no intervention	Members of four guru-led new religious movements (Chinmaya Mission, Mata Amritanandamayi Mission, the Art of Living Foundation, and the Isha Foundation); Pre intervention mean	Daily Spiritual Experiences Scale (DSES; Underwood & Teresi, 2002); Religious Commitment Inventory-10 (RCI-10; Worthington et al., 2003)	Customized Facebook yoga lessons: P, B, R, M, RB, L	3 years; daily spiritual messages + 30 min audio or video yoga lesson 1 time/week, + daily home practice	Increased level of spiritual experiences and religious commitment compared to control group at the end of the intervention; strongest predictor of positive outcomes was regular self-practice	India

				age: 42,4 yrs; 55.8% female					
Park et al., 2016	cross-sectional	N=516, 2 subgroups: Yoga teachers: N=156, Yoga students: N=360	no	Regular yoga practitioners; mean age: 43.9 yrs; 88.9% female	Motives for adopting and maintaining yoga practice	Various forms of hatha yoga	Average number of years of yoga practice: 8.41 for students, 13.16 for teachers; Average amount of yoga practice: 5.5 hours/week for students, 9.03 hours/week for teachers	Primary reasons for adopting yoga practice are exercise, flexibility, and stress relief, and top changed primary reason is spirituality for both students and teachers; spirituality is a reason for maintaining practice	USA
Safara & Ghasemi, 2017*	longitudinal	N=20	N=20, no intervention	Male air traffic controllers in Tehran flight control center; mean age: NR, age between 25-40 yrs; 0% female	Spiritual Intelligence Self-Report Inventory (SISRI; King & DeCicco, 2009), 4 subscales: 1. Critical existential thinking, 2. Production of personal meaning, 3. Transcendental consciousness, 4. Expanded state of consciousness	Yoga exercise	8 weeks; 2.5 hours classes 2 times/week + 1 hour/day home practice (½ hour in the morning and ½ hour in the evening)	All aspects of spiritual intelligence increased in the yoga group compared to the control group	Iran
Scherwitz et al., 2006	longitudinal	N=46	no	two Zen hospice volunteer cohorts, out of them 21 yoga practitioners; mean age: 41 yrs; 76% female	FACIT-Sp (Peterman et al., 2002), 2 subscales: 1. Sense of meaning/peace, 2. Role of faith; Self-Transcendence Survey (Reed, 1991)	Hatha yoga	No yoga intervention (training program for beginning hospice volunteers)	Yoga practitioners have consistently lower fear of death than the group average	USA
Seena et al., 2017	cross-sectional	N=60	N=60, non-yoga practitioners	Regular yoga practitioners; mean age: NR, age between 25-50 yrs; 43.3% female	Spiritual Intelligence Self-Report Inventory (SISRI; King & DeCicco, 2009), 4 subscales: 1. Critical existential thinking, 2. Production of	NR	Number of years of yoga practice: 3 or more years of practice	Higher level of Spiritual Intelligence in all dimensions of Spiritual Intelligence among yoga practitioners compared to control	India

					personal meaning, 3. Transcendental consciousness, 4. Expanded state of consciousness				
Smith et al., 2011*	longitudinal	N=43, 2 subgroups: G1: Integrated Yoga N=33, G2: Yoga-as-exercise N=10	N=26, no intervention	Undergraduate students; mean age: 21.2 yrs; 50.5% female	Herth Hope Scale (HHS; Herth, 1992)	Hatha yoga: P, B, R, M, and homework; G1: Meditation with yamas and niyamas of yogic philosophy, G2: Meditation using mindful breathing	7 weeks; 1-hour classes 2 times/week	Increased level of hope in both yoga groups compared to control	USA
Quilty et al., 2013	cross-sectional	N=604	no	Adults enrolled in a 4-week beginner yoga program (BYP); median age: 35.5 yrs; 86% female	Use of yoga in a real-world setting: perceptions of yoga, motives for yoga practice	Enrolled in BYP of three styles: hatha, kundalini, and ashtanga	Beginners or retuners starting a 4-week program	73% endorse yoga as a spiritual activity; for 37% of the respondents seeking a spiritual experience is a reason to start or return to yoga (the fourth reason after general wellness, physical exercise, and stress management)	USA

Note. NR: not reported; P: posture; B: breathing exercise; R: relaxation; M: meditation; LS: lifestyle advice; RB: reading books, manuals, or other teachings; L: lectures on philosophy; Ma: mantras; FACIT-Sp: Functional Assessment of Chronic Illness Therapy - Spiritual Well-Being Scale; *: study with randomized design

Table 2
Summary of the articles with qualitative approach

**Article**	**Design**	**Sample size (yoga group)**	**Control group(s)**	**Sample condition, age, gender**	**Measurement tool**	**Type of yoga practice**	**Frequency of intervention/previous yoga experience**	**Major findings**	**Origin of the study**
Dittmann & Freedman, 2009; Study 2.	cross-sectional	N=18	no	Regular yoga practitioners from yoga and fitness centers; mean age: NR, age between 23-62 yrs; 100% female	15-minute recorded phone interview: how yoga practice affected spirituality, and how spirituality affected eating and body image attitudes	NR (postural yoga)	Average number of years of yoga practice: 12.1; at least 1 hour/week	Positive changes in sense of spirituality; yoga is a method for spiritual growth	USA
Griera, 2017	longitudinal	N=56	no	Prisoners (time spent in prison M=2.25 yrs); mean age: 38.2 yrs; 16% female	Surveys (N=56) and interviews (N=25) of the impact, meaning, and implications of yoga practice in prison	Holistic program: Kundalini yoga based physical practice, and suggestion for yoga philosophy literature<br>P, B, R, M, RB, Ma	"yoga quarantine": 40 days, 2-3 hours/day; "yoga intensive": 2 months, 2 hours/3 times a week	Yoga is different from other form of movements:<br>1. Divine and transcendent experiences (feeling of freedom, silence, connectedness)<br>2. Brings an expansive spiritual semantic framework of life, it is a means of self-improvement<br>3. Offers a transcendent layer of reality	Europe (Spain)
Hasselle-Newcombe, 2005	cross-sectional	N=188	no, but compared to data of general UK population	Regular Iyengar yoga practitioners; mean age: 47 yrs; 84% female	17 pages long questionnaire: multiple choice, Likert scale questions and qualitative comments on aspects of yoga	Iyengar yoga (postural yoga)	Number of years of yoga practice: between 11 and 15; Average amount of yoga practice: 1 class/week + home	Long-term practitioners are no more likely to have a spiritual interest in their practice than beginners;	Europe (UK)

					practice which are not directly relevant to spirituality, reasons why people begin and continue to practice yoga, perceived effects of yoga		practice 3–5 hours/week	yoga gives sense of meaning to life in 85% of practitioners;<br>83% of yoga practitioners have a spiritual life compared to 45% of the general UK population;<br>concept of God associated with spirituality and not religiosity;<br>spirituality: experience of 'imminence', inward looking, mundane, but very personal experiences of holiness	
Lewis, 2008	cross-sectional	N=9	no	Regular vinyasa yoga practitioners; mean age: NR; gender: NR	Loosely structured interviews with a focus on motivational factors for yoga practice	Vinyasa yoga (postural yoga)	NR	Enmeshed social, psychological, physical and spiritual meanings and motivations to continue yoga practice;<br>motivation changes with time: originally to achieve the perfect 'yoga body', later deeper mental and spiritual motivation	USA

Ness & Briles, 2016	cross-sectional	N=11	no	Yoga instructors; mean age: NR; 82% female	In-depth, semi-structured interviews about the traditional spirituality of yoga	Trained yoga teachers in various hatha yoga styles	NR	Lack of spirituality in westernized yoga; importance of meditation and yoga philosophy for spiritual progress; practice is the key to entering into yoga spirituality	USA
Pandya, 2018; Part 2.	longitudinal	N=19	no	Auroville spiritual community residents; Post intervention mean age: 44 yrs; 31,6% female	In-depth interview: open-ended questions about life and existence, aspirations, fears and anxieties, and spiritual experiences	Integral yoga of Sri Aurobindo<br>LS	10 years, NR	Increased level of existential thinking, spiritual inclinations and integrative worldview after 10 years of residence; important aspects: philosophical teachings, consecration, meditation, prayer, and social support	India
Wahlström et al., 2018*	longitudinal	N=12	N=44, relaxation group N=44, only standard treatment	Patients with symptomatic paroxysmal atrial fibrillation; mean age: 63.5 yrs; 41.6% female	Individual semi-structured interviews on how participants experienced therapeutic yoga	MediYoga (therapeutic yoga derived from Kundalini yoga)<br>P, R, M	12 weeks, weekly 1 hour + encouraged to perform MediYoga at home with CD-record	Yoga participants' perceptions and experiences: 1. A sense of existence and presence – experience of more inner peace, 2. Increased awareness of connection of physical and mental functions	Europe (Sweden)

Note. NR: not reported; P: posture; B: breathing exercise; R: relaxation; M: meditation; LS: lifestyle advice; RB: reading books, manuals, or other teachings; Ma: mantras; *: study with randomized design.

Table 3

Summary of the articles between 2020-2021 (all with quantitative approach)

**Article**	**Design**	**Sample size (yoga group)**	**Control group(s)**	**Sample condition, age, gender**	**Measurement tool**	**Type of yoga practice**	**Frequency of intervention/previous experience with yoga**	**Major findings**	**Origin of the study**
Bringmann et al., 2021	longitudinal	N=20	no	Patients with a current diagnosis of a mild or moderate depressive episode; mean age: NR; 88% female	Aspects of Spirituality (ASP; Büssing et al., 2010), 4 subscales: 1. Religious orientation: prayer/trust in God, 2. Search for insight/wisdom, 3. Conscious interactions/compassion, 4. Transcendence conviction	Meditation Based Lifestyle Modification (MBLM) - a complex mind-body intervention: P, B, R, M LS, RB, L (yama, niyama emphasized), Ma	8 weeks, weekly 3-hour class + 45 min daily home practice recommended	Increase in Search for Insight/wisdom, but no changes in the other 3 subscales at the end of the intervention	Europe (Germany)
Bryan et al., 2021	longitudinal	N=8	no	Cancer survivors; mean age: 61 yrs; 87.5% female	FACIT-Sp (Peterman et al., 2002), 3 subdomains: meaning, peace, and faith; 2 open-ended questions	Gentle yoga and mindfulness meditation: P, B, R, M	10 weeks; 45 min classes 2 times/week	Increased spiritual well-being at the end of the intervention; increased levels of meaning and faith, but no changes in peace; reported spiritual perceptions: peaceful, tranquil, opportunity for prayer, spiritually connected	USA
Büssing et al., 2021	cross-sectional	N=901	no	Regular yoga practitioners; mean age: 49 yrs; 89% female	Interpretation of yoga; Yama/Niyama Questionnaire (YaNiQ; Büssing et al., 2021)	Various forms of yoga: 88% Yoga Vidya	Average number of years of yoga practice: 13, Average amount of yoga practice 55% several times/week;	Yoga as a spiritual path for 78%; Participants' perspectives on their practice whether it is seen as gymnastics or	Europe (Germany)

							31% daily; 11% once/week, 3% less than once/week	spiritual path influence yama/niyama scores: participants regarding yoga as a spiritual path have higher scores for (1) Devotion/Self-Reflection/Contentment, (2) Restrain/Truthfulness/ Self-Discipline, and (3) Non-Possessiveness, but not for (4) Non-Harming	
Cartwright et al., 2020	cross-sectional	N= 2434	no	Adult UK residents practicing yoga within the previous 12 months; mean age: 48.7 yrs; 87.3% female	Motivation for yoga practice	Various forms of hatha yoga	Average number of years of yoga practice: 13.9; Average amount of yoga practice: 5.52 hours/week	Initial reasons were general wellness (39%), fitness (19%) and flexibility (8.5%); most notable change in spirituality over time: recent spiritual motivation 21%	Europe (UK)
Csala et al., 2020	longitudinal	N=50, 2 subgroups: Sport group (physical aspect emphasized): N=27, Spiritual group (philosophical and spiritual contents included): N=23	N=34, no intervention	Beginner female students; mean age: 22 yrs, 100% female	Spiritual Connection Questionnaire (SCQ-14; Wheeler and Hyland, 2008)	Beginner hatha yoga; Sport group: P, B, R; Spiritual group: P, B, R, Phi, Ma	10 weeks; weekly 1.5 hours class, no home practice	No differences between the two yoga groups; Increased spiritual connection in the merged yoga group compared to control at the end of the intervention	Europe (Hungary)

Park et al., 2020	interventi onal	N=144	no	Regular yoga practitioners (average number of years of yoga practice: 7.1); mean age: 40 yrs; 78% female	Psychological resources: Self-Transcendence Subscale of the Temperament and Character Inventory (STS; MacDonald & Holland, 2002); Peace subscale of FACIT-Sp (Canada et al., 2008)	11 forms of hatha yoga: included components measured by Essential Properties of Yoga Questionnai re (EPYQ; Park et al., 2018)	1-hour long single yoga session	Increased self-transcendence and spiritual peace after yoga class independently of style; both changes in self-transcendence and spiritual peace favorably correlated with changes in emotions; higher levels of warmth and friendliness of the yoga teacher correlate with increased self-transcendence	USA

Note. NR: not reported; P: posture; B: breathing exercise; R: relaxation; M: meditation; LS: lifestyle advice; RB: reading books, manuals, or other teachings; Phi: philosophy, L: lectures on philosophy; Ma: mantras; FACIT-Sp: Functional Assessment of Chronic Illness Therapy - Spiritual Well-Being Scale.
